# High Fidelity Cryopreservation and Recovery of Primary Rodent Cortical Neurons

**DOI:** 10.1523/ENEURO.0135-18.2018

**Published:** 2018-09-27

**Authors:** Sara S. Parker, Aubin Moutal, Song Cai, Sambamurthy Chandrasekaran, Mackenzie R. Roman, Anita A. Koshy, Rajesh Khanna, Konrad E. Zinsmaier, Ghassan Mouneimne

**Affiliations:** 1 Department of Neuroscience, College of Science, University of Arizona, Tucson, AZ 85724; 2 Department of Pharmacology, College of Medicine, University of Arizona, Tucson, AZ 85724; 3 Bio5 Institute, University of Arizona, Tucson, AZ 85724; 4 Department of Molecular and Cellular Biology, College of Science, University of Arizona, Tucson, AZ 85724; 5 Departments of Neurology, Cellular and Molecular Medicine, and Immunobiology, College of Medicine, University of Arizona, Tucson, AZ 85724; 6 Departments of Neuroscience, Pharmacology, and Anesthesiology, Colleges of Science and Medicine, University of Arizona, Tucson, AZ 85724; 7 Departments of Neuroscience and Molecular and Cellular Biology, College of Science, University of Arizona, Tucson, AZ 85724; 8 Department of Cellular and Molecular Medicine, College of Medicine, University of Arizona, Tucson, AZ 85724

**Keywords:** cryopreservation, primary neuron culture

## Abstract

Cell cryopreservation improves reproducibility and enables flexibility in experimental design. Although conventional freezing methodologies have been used to preserve primary neurons, poor cell viability and reduced survival severely limited their utility. We screened several high-performance freezing media and found that CryoStor10 (CS10) provided superior cryoprotection to primary mouse embryonic cortical neurons compared to other commercially-available or traditional reagents, permitting the recovery of 68.8% of cells relative to a fresh dissection. We characterized developmental, morphometric, and functional indicators of neuron maturation and found that, without exception, neurons recovered from cryostorage in CS10 media faithfully recapitulate *in vitro* neurodevelopment in-step with neurons obtained by fresh dissection. Our method establishes cryopreserved neurons as a reliable, efficient, and equivalent model to fresh neuron cultures.

## Significance Statement

In this study, we have optimized and validated a methodology for the efficient cryopreservation of primary embryonic rodent neurons, yielding cells which are developmentally and functionally similar to fresh neurons. This approach serves as an effective alternative to regularly obtaining neurons by fresh dissection and thus frees researchers from substantial labor and animal husbandry costs, enables flexibility in experimental timelines, and conserves animals.

## Introduction

Primary neuronal cultures derived from rodents are critical experimental model systems in cellular and molecular neurobiology. Methods pioneered by Banker and others permit the culturing of various neuronal populations from central and peripheral nervous systems (Banker, 1998). These culturing techniques made possible the study of cell-intrinsic pathways important for neuronal morphogenesis and development, and enabled observations of cellular and molecular processes at the neuron-neuron or neuron-glia interface. However, the requirement of fresh tissue, frequently from an embryonic or neonatal source, remains a major limiting factor in the practicality and accessibility of primary neuron culture.

After tissue dissociation and subsequent plating, cultured neurons undergo a defined differentiation program spanning several weeks. The inherent inflexibility of this developmental program necessitates frequent dissections and continuous culturing, resulting in labor intensive and often wasteful over-culturing to meet experimental requirements. The use of stem cells, or cell lines with neuron-like properties can eliminate the need for primary tissue, but introduces new caveats and limitations (Gordon et al., 2013; Marx, 2016). Thus, despite their inconvenience and cost, dissociated rodent neuron cultures remain an essential model in neuroscience.

Cryopreservation halts biochemical and cellular processes, thus allowing the indefinite storage of viable cells (Pegg, 2015). Cryostorage and recovery of viable, healthy neurons would offer a practical alternative to obtaining neurons by fresh dissection. Timed pregnancies are often unpredictable sources of embryonic tissue, while a stock of cryopreserved neurons guarantees their steady availability for experiments. Neurons can be recovered on-demand, permitting time courses not feasible with timed pregnancies. Considering the ease of sharing frozen cells, cryopreserved primary neurons could serve as an alternative to sharing mice across institutions, facilitating collaboration. Importantly, the conservation of otherwise extra cells after tissue dissection and dissociation facilitates full utilization of valuable resources and reduces the use of animals. As such, cryopreservation would improve the efficiency of laboratories with low-volume neuron usage.

Despite these benefits, the adoption of neuronal cryopreservation methods has been limited (Paynter, 2008). Pioneering studies found that neuronal cells could be frozen in “hibernation media” supplemented with cryoprotectant, which on revival yielded neurons that appeared morphologically and physiologically normal (Kawamoto and Barrett, 1986; Mattson and Kater, 1988; Petite and Calvet, 1995; Otto et al., 2003; Quasthoff et al., 2014). However, despite optimization of freezing parameters and media composition, the viability and long-term survival of cryostored neurons was consistently low using these methods (Higgins et al., 2011; Robert et al., 2016), rendering neuronal cryopreservation an inefficient practice.

Cryopreservation methods were originally devised to control the major causes of cryopreservation-related cell mortality, namely, intracellular ice formation (Pegg, 2015). However, traditional freezing media formulations provide inadequate protection against the myriad of additional physical and chemical insults inflicted by cryostorage. Freezing media comprised of base culture media, serum, and cryoprotectant, such as DMSO, are isotonic and promote osmotic stress during freezing, thereby reducing viability and yield (Hawkins et al., 2017). Moreover, biochemical activity persists until cells vitrify into a non-ice solid at approximately -140°C, permitting the accumulation of energy imbalances and free radicals during the slow-freezing step of cryopreservation (Baust et al., 2016). These stressors can activate apoptotic and necrotic pathways, promoting cryopreservation-induced delayed-onset cell death (CIDOCD), occurring up to 48 h after plating (Baust et al., 2002). Recent studies demonstrated that the remnants of cryodamage can persist for the lifetime of the culture through epigenetic modifications and genetic drift, affecting the long-term survival and performance of cells and compromising experimental reproducibility (Chatterjee et al., 2017). The expansion of cell therapy into the clinic has demanded commensurate improvements in cryobiology methods for the storage of stem and primary cells (Baust et al., 2016). Several commercially-available, specialty freezing, media have been designed to improve the recovery and fidelity of primary cells after cryostorage. Until now, the applicability of these reagents for primary neuron cryopreservation has not been tested.

We identified a high-performing freezing media, CryoStor10 (CS10; BioLife Solutions), and optimized its use for the cryopreservation of dissociated primary neurons. We demonstrated its suitability for the efficient recovery of healthy embryonic cortical neurons. Applying a rigorous validation strategy to assess development, morphogenesis, synaptogenesis, and electrophysiological performance, we show that neurons recovered from cryostorage and those obtained from a fresh dissection are indistinguishable. Our data establish the utility of cryopreserved neurons for low-density dissociated cultures in imaging and electrophysiology experiments.

## Materials and Methods

### Animals

All animal procedures were performed in accordance with the University of Arizona animal care committee’s regulations. Pregnant C57BL/6 mice were killed by CO_2_ asphyxiation on embryonic day 17. Cortical and hippocampal neurons were isolated from embryos of either sex and cultured by methods described previously (Kaech and Banker, 2006).

### Cell culture

Culturing surfaces were prepared by coating overnight with 0.001% poly-L-lysine (MilliporeSigma, P4707, diluted in H_2_O 1:10), washed three times for ten minutes each with water, and transferred to Plating Media, comprised of MEM (Corning, 10-010CV), 0.6% D-glucose (MilliporeSigma), 10% horse serum (Thermo Fisher Scientific, 26050-088). Neurons were counted and seeded in Plating Media at appropriate densities: 0.67 × 10^3^ cells/mm^2^ for imaging and electrophysiology, and 2.80 × 10^3^ cells/mm^2^ for glutamate release assay and RNA samples. Two to four hours after plating, cells received a full media exchange to Neurobasal Maintenance Media, comprised of Neurobasal base media (Thermo Fisher, 21103049), 2% B_27_ (Thermo Fisher, A3582801), 1% L-glutamine (Corning, 25-005CI), and 1% penicillin-streptomycin (Corning, 30-002CI). For all subsequent media changes, Neurobasal Maintenance Media without B_27_ were conditioned overnight by glial cells maintained in a separate culture. B_27_ was added fresh at the time of media change. On day *in vitro* (DIV)4, neurons received a half-volume change of conditioned Neurobasal Maintenance Media plus 5 μM cytosine arabinoside (AraC, MilliporeSigma, C6645) to curb glial proliferation. One-third volume media exchanges with conditioned Neurobasal Maintenance Media occurred every 3–4 d thereafter. All experiments comparing freshly dissected and cryostored neurons, except for paired sample experiments and electrophysiological recordings, were plated within 30 min of each other to ensure synchronous development. All viability and validation experiments using cryostored neurons were performed on aliquots stored between three and 90 d in liquid nitrogen, unless indicated otherwise.

### Freezing and thawing neurons

CryoStor CS10 and CS5 (BioLife Solutions), and Synth-a-Freeze (SAF; Thermo Fisher) were used in accordance with manufacturer’s instructions or as previously described (Newman and Kaur, 2015).

To freeze, dissociated cells from a fresh dissection were pelleted, resuspended in ice-cold freezing media at a density of 6 × 10^6^ cells/ml, and aliquoted into cryovials on ice. Each aliquot contained 1.5 × 10^6^ cells. Cryovials were placed in an isopropanol cell freezing container pre-chilled to 4°C, stored at -80°C for 2 d, and then transferred to the vapor phase of liquid nitrogen for long-term storage.

To thaw, to reduce shear stress during handling, cells were transferred and resuspended using a P1000 tip cut to widen the diameter to ∼2 mm. Cryovials were rapidly thawed in a 37°C water bath until a small ice crystal remained. Cells were then gently resuspended by drop-wise addition of warm Plating Media to the cryovial with periodic swirling. This volume was gently transferred to a conical tube containing 10× volume warm Plating Media. The cell suspension was pelleted by centrifugation at 150 × *g* for 5 min and resuspended in 1 ml fresh Plating Media by pipetting ∼15 times with a cut P1000 to break up cell clumps. Cells were counted using Trypan blue and a hemocytometer and plated as required.

### Viability and survival

Percentage viability and percentage recovery of cells were determined immediately post-thaw using Trypan blue exclusion and a hemocytometer. Viable cell yield was calculated with (percentage viability × percentage recovered)/100), as previously described (Higgins et al., 2011). Cell survival at DIV3 was determined using LIVE/DEAD Viability/Cytotoxicity kit (Thermo Fisher) per manufacturer’s instructions.

### Microscopy and software

Widefield fluorescence imaging was performed on a Nikon Eclipse T*i* (Nikon Instruments Inc.) inverted microscope configured with a Chroma DAPI/FITC/Cy3/Cy5 filter (Chroma Technologies, 89400). Samples were imaged with a 20× Plan Apo 0.75 NA, 40× Plan Fluor 1.30 NA oil-immersion, or 100× Apo TIRF 1.49 NA oil-immersion objective. Image acquisition was performed using a Hamamatsu ORCA-Flash 4.0 V2 cMOS camera (Hamamatsu Photonics). Images were acquired in a 2 × 2 or 4 × 4 grid and stitched using 10% overlap to create a composite image using the Large Stitch Image acquisition setting in NIS Elements. All analysis and figure preparation was performed using ImageJ/FIJI (RRID:SCR_002285; Schindelin et al., 2012).

### Plasmids and lentivirus

Lentiviral transfer plasmid pCIG3 (Addgene #78264, a gift from Felicia Goodrum; Caviness et al., 2014) was modified to express a blasticidin resistance gene in place of GFP. TurboRFP was subcloned into this backbone using AgeI and PstI to generate pLenti TurboRFP BlastR for cytoplasmic TurboRFP expression. Lentiviral transfer plasmid pLenti Lifeact-mRuby2 BlastR was generated by Multisite Gateway recombination of pENTR CMVie-Lifeact-mRuby2 L1-R5 (Addgene #84389), pMuLE ENTR MCS L5-L2 (Addgene #62085), and pLenti Dest BlastR (Addgene #84574). pLenti TurboRFP BlastR and pLenti Lifeact-mRuby2 BlastR have been deposited to Addgene under #102343 and #84384, respectively.

For exogenous protein expression in cultured neurons, second generation lentiviral particles were generated by PEI transfection of 293T cells as previously described (Yang et al., 2017) with transfer plasmid, pMD2.G, and psPAX2 (Addgene #12259, #12260, gifts from Didier Trono). At 24 and 48 h post-transfection, 293T media containing lentiviral particles was collected, combined, filtered, and added directly to neuron cultures without polybrene at the time of plating. pLenti TurboRFP BlastR’s functional titer was 5.25 × 10^5^ TU/ml, used at 0.5 MOI. pLenti Lifeact-mRuby2 BlastR’s functional titer was 3.01 × 10^5^ TU/ml, used at 2.5 MOI. Antibiotic selection was not used in neuronal transductions.

### Immunofluorescence

Cells were grown on 0.001% poly-L-lysine-coated glass coverslips (Carolina Biological Supply, #633029). Cells were fixed for 15 min at 37°C in warm 4% paraformaldehyde (diluted from 16% in PBS, Electron Microscopy Sciences) plus 4% sucrose in PBS. Coverslips were blocked for 30 min in 10% goat serum (Thermo Fisher, 16210-072) plus 0.1% Triton X-100 (MilliporeSigma) in PBS and incubated overnight at 4°C with primary antibody diluted in 3% goat serum plus 0.1% Triton X-100 in PBS, and similarly incubated for 45 min at room temperature with secondary antibodies or phalloidin. Coverslips were mounted in Prolong Gold Antifade mounting media (Thermo Fisher). Primary antibodies, their usage, and source are given in Table 1. Secondary antibodies are as follows: goat anti-mouse/rabbit Alexa Fluor 405, 488, 568, or 647 (Thermo Fisher, 1:200), and goat anti-chicken Alexa Fluor 488 (Thermo Fisher, 1:200). Fluorescently-conjugated phalloidin was used to label F-actin (Alexa Fluor 488 or Alexa Fluor 568 phalloidin, Thermo Fisher).

**Table 1.** Primary antibody source and usage

Target name	Source	RRID	Dilution
Iba1	Rb polyclonal, Wako (019-19741)	RRID:AB_839504	1:500
S100	Rb polyclonal, Dako (Z0311)	RRID:AB_10013383	1:400
Olig2	Ms monoclonal, MilliporeSigma (MABN50, 211F1.1)	RRID:AB_10807410	1:500
β-III-tubulin	Rb monoclonal, Cell Signaling (5568, D71G9)	RRID:AB_10694505	1:200
β-III-tubulin	Ms monoclonal, MilliporeSigma (MAB1637, TU-20)	RRID:AB_2210524	1:200
Tau	Ms monoclonal, MilliporeSigma (MAB3420, PC1C6)	RRID:AB_94855	1:750
MAP2	Rb monoclonal, Cell Signaling (8707, D5G1)	RRID:AB_2722660	1:200
PSD95^1^	Ms monoclonal, UC Davis/NIH NeuroMab Facility (75-028 K28/43)	RRID:AB_2292909	1:100
PSD95^2^	Ms monoclonal, Novus Biologicals (NB300-556, 6G6-1C9)	RRID: AB_2092366	1:500
Synaptophysin	Ck polyclonal, Synaptic Systems (101 006)	RRID:AB_2722661	1:500
Gephyrin	Rb polyclonal, Alomone (AIP-005)	RRID:AB_2722662	1:500

Rb: rabbit, Ms: mouse, Ck: chicken.

^1^ used in Figure 6.

^2^ used in Figure 9.

### Morphometric and Sholl analysis

Neurons were fixed and immunolabeled for Tau and MAP2 on DIV4 and DIV7 after plating. Individual neurons with clearly defined arbors, and expressing pLenti TurboRFP BlastR (DIV7 only) were traced using the FIJI plug-in Simple Neurite Tracer (Longair et al., 2011). Tracings were used to determine the number and length of primary neurites (branches emanating from the soma), and length of the axon (the longest Tau-positive process). Sholl analysis was performed on binary masks of traced neurons generated by the Fill Out function in Simple Neurite Tracer. The center of analysis was defined by a point ROI at the cell soma. Binary masks were loaded into the FIJI plug-in Sholl analysis (Ferreira et al., 2014), and batch processed by setting a maximum radius of 2000 μm, a continuous step size of radius 0, and fitting to an 8th degree polynomial using the Log-log method. Sholl profiles of neurons at DIV4 or DIV7 are presented as mean ± SD. Additional calculated metrics presented include the critical value, critical radius, enclosing radius, and Sholl regression coefficient.

### Assessment of synapse formation

Cells were fixed and immunolabeled on DIV12, DIV14, and DIV16 after plating. Secondary dendrites of neurons expressing pLenti Lifeact-mRuby2 BlastR were analyzed. Images were acquired with the 100× objective in a 2 × 2 grid and stitched into a composite image using the Large Stitch Image acquisition setting in NIS Elements. At each position, Z-stacks of 0.2 μm step and 1- to 2-μm depth were acquired to create a single focused image using the Extended Depth of Focus (EDF) feature in NIS Elements. The Blind Deconvolution algorithm in NIS Elements was applied with two iterations to generate a final processed image. Protrusion density was determined by quantifying the number of PSD95-positive protrusions as dendritic spines or PSD95-negative protrusions as filopodia. These values were presented as counts per length of dendrite. Synapse type was quantified by counting the number of PSD95- or gephyrin-positive puncta that coincided with synaptophysin as excitatory or inhibitory synapses, respectively.

### Reverse transcription quantitative PCR (RT-qPCR)

Total cellular RNA was isolated from DIV12 neurons using Direct-zol RNA MiniPrep kit (Zymo Research Corp., R2060) and TRIzol (Thermo Fisher, 15596026), according to manufacturer’s instructions, and including the optional DNase on-column digestion to eliminate genomic DNA. cDNA was synthesized from 1000 ng of input RNA using qScript cDNA Synthesis kit (Quantabio, 95047). RT-qPCR reactions were run in duplicate using an ABI 7500 Fast Real-Time PCR System (Applied Biosystems) with Apex GREEN Low ROX Master Mix (Genesee, 42-118). Primer sequences and sources are given in Table 2. Primer pairs were confirmed to have 85–110% efficiency based on the slope of the standard curve from a cDNA dilution series. C_T_s were normalized to the mean C_T_ of two housekeeping genes, *Eef1a1* and *Rpl29*. Relative copy number was determined using the comparative C_T_ method (2^-ΔCT^; Schmittgen and Livak, 2008), and deriving a value relative to 1 × 10^6^ copies of *Eef1a1*.

**Table 2.** RT-qPCR primer sequences

Target name	Sequence	Direction	Source
Eef1a1	CAACATCGTCGTAATCGGACA	Fwd	PrimerBank, Harvard
	GTCTAAGACCCAGGCGTACTT	Rev	
Rpl29	CAAGTCCAAGAACCACACCAC	Fwd	PrimerBank, Harvard
	GCAAAGCGCATGTTCCTCAG	Rev	
Pax6	TACCAGTGTCTACCAGCCAAT	Fwd	PrimerBank, Harvard
	TGCACGAGTATGAGGAGGTCT	Rev	
Emx1	GAAGAATCACTACGTGGTGGG	Fwd	Terrigno et al. (2008)
	CCGTTTGTATTTTGTCCTCCGA	Rev	
Emx2	GGCTAGAGCACGCTTTTGAG	Fwd	Terrigno et al. (2008)
	CACCGGTTAATGTGGTGTGT	Rev	
Mtap2	CTCCTCGCAGGGGTGTATCA	Fwd	PrimerBank, Harvard
	GTCCGTCGTGCTGAAGAGA	Rev	
Tbr1	CGCCCTCCTCCATCAAATCCATCG	Fwd	Terrigno et al. (2008)
	GCAGTTCTTCTCGCAGTCCCGC	Rev	
Reln	TTACTCGCACCTTGCTGAAAT	Fwd	PrimerBank, Harvard
	CAGTTGCTGGTAGGAGTCAAAG	Rev	
Sat2b	GCCGTGGGAGGTTTGATGATT	Fwd	PrimerBank, Harvard
	ACCAAGACGAACTCAGCGTG	Rev	
Cux1	TGACCTGAGCGGTCCTTACA	Fwd	PrimerBank, Harvard
	TGGGGCCATGCCATTTACATC	Rev	
Lhx9	TCCAAAACGCACGAGCCAA	Fwd	Terrigno et al. (2008)
	CAGGTCTGTTAAAGTGGTCGC	Rev	
Lmo3	ACACGAAGGCTAACCTTATCCT	Fwd	Terrigno et al. (2008)
	AGTTTCCCGTTACACCAAACAG	Rev	
Gap43	TGGTGTCAAGCCGGAAGATAA	Fwd	PrimerBank, Harvard
	GCTGGTGCATCACCCTTCT	Rev	
Slc17a6	CTGAGAAGAAGGCTCCGCTAT	Fwd	PrimerBank, Harvard
	ATGCCGAAGGATATGCAGAAG	Rev	
Slc32a1	ACCTCCGTGTCCAACAAGTC	Fwd	PrimerBank, Harvard
	TCAAAGTCGAGATCGTCGCAG	Rev	
Tubb3	GCCAAGTTCTGGGAGGTCAT	Fwd	PrimerBank, Harvard
	GGGCACATACTTGTGAGAGGA	Rev	
Gfap	ACCAGCTTACGGCCAACAG	Fwd	PrimerBank, Harvard
	CCAGCGATTCAACCTTTCTCT	Rev	
Bcl11b	CCCGACCCTGATCTACTCAC	Fwd	PrimerBank, Harvard
	GGAGGTGGACTGCTCTTGT	Rev	
Nes	CCCCTTGCCTAATACCCTTGA	Fwd	PrimerBank, Harvard
	GCCTCAGACATAGGTGGGATG	Rev	
Foxg1	CACTTTGAGTTACAACGGGACC	Fwd	PrimerBank, Harvard
	CGAGTTTTGAGTCAACACGGA	Rev	

### Quantification of glutamate release

Glutamate levels were measured using an Amplex Red Glutamic Acid/Glutamate Oxidase Assay kit (Thermo Fisher, A12221) as described previously (Wang et al., 2010). Briefly, neurons were cultured in a 12-well plate in triplicate until DIV14. Samples were collected before stimulation, after 15-min depolarization in a buffer containing 90 mM KCl, and poststimulation. All wells were treated with 500 μM DL-threo-β-benzyloxyaspartate (TBOA; Tocris) to prevent glutamate reuptake. Control wells were incubated with the calcium channel blocker cadmium chloride to prevent glutamate release. Samples were incubated with Amplex Red reagent per manufacturer’s instructions at 37°C for 30 min, and measured with a BioTek Synergy H1 Hybrid Multi-Mode microplate reader (BioTek) at excitation/emission 530 nm/590 nm. Fluorescence measurements were normalized to a blank well, and glutamate concentrations were calculated using a standard curve ranging from 0.5 to 30 μM. Total glutamate released was calculated by summing the basal, stimulated, and poststimulation glutamate concentrations for each experimental condition.

### Whole-cell patch clamp electrophysiology

Electrodes were pulled from thin-walled borosilicate glass capillaries with a P-97 electrode puller (Sutter Instruments) such that final electrode resistances were 1–3 MΩ when filled with internal solutions. The internal solution contained 140 mM KCl, 10 mM NaCl, 1 mM MgCl_2_, 1 mM EGTA, 10 mM HEPES, and 1 mM ATP-Mg (pH 7.2, ∼295 mOsm/l), and external solution contained 154 mM NaCl, 5.6 mM KCl, 2 mM CaCl_2_, 2 mM MgCl_2_, 1 mM glucose, and 10 mM HEPES (pH 7.2, ∼305 mOsm/l). To assess excitability, spontaneous action potential (AP) firing was recorded for 2 min from neurons with a resting membrane potential more hyperpolarized than -40 mV and stable baseline recordings. Spontaneous recordings were performed by setting the recording mode to current clamp without injecting any current. APs were evoked by serial current injection steps with an increment of 50 pA for 50 ms. The first AP evoked was used for analysis of wave form parameters. Activity was recorded using an EPC 10 Amplifier and analyzed with a Fitmaster (HEKA Elektronik). AP traces were analyzed using Clampfit 10.7 (Molecular Devices). Capacitive artifacts were fully compensated, and series resistance was compensated by ∼70%. Recordings made from cells with greater than a 5-mΩ shift in series resistance compensation error were excluded. All experiments were performed at room temperature (∼23°C). The currents were filtered at 5 kHz and sampled at 2 kHz using a Patchmaster (HEKA Elektronik).

### Calcium imaging

Neurons were loaded at 37°C with 3 μM Fura-2 AM (Thermo Fisher, F-1221) and 0.02% pluronic acid (Thermo Fisher, P3000MP) for 30 min (*K*_d_ = 25 μM, λ_ex_ 340, 380 nm/λ_emi_ 512 nm) to follow changes in intracellular calcium ([Ca^2+^]_c_) in Tyrode’s solution (at ∼310 mOsm) containing 119 mM NaCl, 2.5 mM KCl, 2 mM MgCl_2_, 2 mM CaCl_2_, and 25 mM HEPES; pH 7.4 and 30 mM glucose. All calcium imaging experiments were done at room temperature (∼23°C). Baseline was acquired for 1 min followed by a 15-s stimulation with either Tyrode’s solution containing either 200 µM glutamate, 100 µM glycine, 50 µM NMDA + 10 µM D-serine, or 90 mM KCl excitatory solution (at ∼310 mOsm) comprised of 32 mM NaCl, 90 mM KCl, 2 mM MgCl_2_, 2 mM CaCl_2_, and 25 mM HEPES; pH 7.4 and 30 mM glucose. Fluorescence imaging was performed with an inverted microscope, Nikon Eclipse T*i*-U (Nikon Instruments Inc.), using objective Nikon S Plan Fluor ELWD 20 × 0.45 NA and a Photometrics cooled CCD camera CoolSNAP ES^2^ (Photometrics) controlled by NIS Elements software. The excitation light was delivered by a Lambda-LS system (Sutter Instruments). The excitation filters (340 ± 5 and 380 ± 7 nm) were controlled by a Lambda 10-2 optical filter change (Sutter Instruments). Fluorescence was recorded through a 505-nm dichroic mirror at 535 ± 25 nm. To minimize photobleaching and phototoxicity, the images were taken every 10 s during the time course of the experiment using the minimal exposure time that provided acceptable image quality. The changes in [Ca^2+^]_c_ were monitored by following the ratio of F_340_/F_380_, calculated after subtracting the background from both channels.

### Statistics

Statistical analyses were performed using Prism 7 (GraphPad). Data sourced from a minimum of three independent biological replicates unless indicated otherwise. For continuous variables, data are presented as a scatterplot, or a box-and-whisker plot of individual data points pooled for all three biological replicates along with the mean value calculated within each biological replicate. Biological replicates are denoted with symbols (○◻△◇). Where indicated, mean value per biological replicate was used to make statistical inferences. Normality tests were performed to determine data distribution before choosing parametric or nonparametric tests. Pearson’s correlation coefficient was used to determine relationship between viability and days in cryostorage. Statistical comparison between mean Sholl profiles or RNA copy number were made by unpaired parametric two-tailed *t* test with Holm–Sidak correction for multiple comparisons, generating a multiplicity adjusted *p* value. Each occurrence of potential Type II error is discussed. Statistical significance was set at α < 0.05. Additional details given in Table 3.

**Table 3.** Statistics table

	Figure	Data structure	Test	CI of difference of mean/median	*p* value		
a	2*B*, Fresh v CS10	Normal	Unpaired parametric two-tailed *t* test	-0.2311 to 0.1168	0.4131	** **	** **
b	2*B*, Fresh v CS5	Normal	Unpaired parametric two-tailed *t* test	-0.4476 to -0.1619	0.0041	** **	** **
c	2*B*, Fresh v SAF	Normal	Unpaired parametric two-tailed *t* test	-0.6306 to -0.3999	0.0002	** **	** **
d	2*B*, Fresh v 50:40:10	Normal	Unpaired parametric two-tailed *t* test	-0.5666 to -0.3437	0.0003	** **	** **
e	2*E*, Fresh v CS10	Normal	Unpaired parametric two-tailed *t* test	4.188 to 22.27	0.0153		
f	2*E*, Fresh v CS5	Normal	Unpaired parametric two-tailed *t* test	4.207 to 46.59	0.0292		
g	2*E*, Fresh v SAF	Normal	Unpaired parametric two-tailed *t* test	34.23 to 78.22	0.0021		
h	2*E*, Fresh v 50:40:10	Normal	Unpaired parametric two-tailed *t* test	*19.57 to 78.16*	0.0098	*U*	
i	2*F*, Fresh v CS10	Nongaussian	Mann–Whitney *U* test	-2.87 to 0.3846 (median)	0.4000	2	
j	2*F*, Fresh v CS5	Nongaussian	Mann–Whitney *U* test	-2.22 to 0.3846 (median)	0.9999	4	
k	2*F*, Fresh v SAF	Nongaussian	Mann–Whitney *U* test	-3.76 to 0.3846 (median)	0.9999	4	
l	2*F*, Fresh v 50:40:10	Nongaussian	Mann–Whitney *U* test	-1.11 to 0.3846 (median)	0.4000	2	** **
						*n*	adj *p* value
m	3	Normal	Unpaired parametric *t* test, multiple comparisons using Holm–Sidak	N/A	0.0050- 0.8389	18 genes	0.0857 -0.9999
n	4*C*, DIV4	Normal	Unpaired parametric two-tailed *t* test	-1.982 to 2.565	0.7397		
o	4*C*, DIV7	Normal	Unpaired parametric two-tailed *t* test	-1.337 to 3.233	0.3135		
p	4*D*, DIV4	Normal	Unpaired parametric two-tailed *t* test	-12.02 to 4.785	0.2980		
q	4*D*, DIV7	Normal	Unpaired parametric two-tailed *t* test	-17.28 to 11.79	0.6278		
r	4*E*, DIV4	Normal	Unpaired parametric two-tailed *t* test	-29 to 78.07	0.2601		
s	4*E*, DIV7	Normal	Unpaired parametric two-tailed *t* test	-119.5 to 133.5	0.8870	** **	** **
t	4*F*	Normal	Paired parametric two-tailed *t* test	-2.378 to 0.8645	0.1824	** **	** **
u	4*G*	Normal	Paired parametric two-tailed *t* test	-15.4 to 6.631	0.2288	** **	** **
v	4*H*	Normal	Paired parametric two-tailed *t* test	-121.6 to 129	0.9097	** **	** **
						*n*	adj *p* value
w	5*D*	Normal	Unpaired parametric *t* test, multiple comparisons using Holm–Sidak	N/A	0.0018- 0.9973	1207 radii	0.8864 -0.9999
x	5*E*	Normal	Unpaired parametric *t* test, multiple comparisons using Holm–Sidak	N/A	0.0128- 0.9974	1610 radii	>0.9999
y	5*F*, DIV4	Normal	Unpaired parametric two-tailed *t* test	-40.41 to 38.08	0.9382		
z	5*F*, DIV7	Normal	Unpaired parametric two-tailed *t* test	-125.3 to 133.2	0.9367		
aa	5*G*, DIV4	Normal	Unpaired parametric two-tailed *t* test	-2.537 to 1.095	0.3320		
ab	5*G*, DIV7	Normal	Unpaired parametric two-tailed *t* test	-1.448 to 1.8464	0.7453		
ac	5*H*, DIV4	Normal	Unpaired parametric two-tailed *t* test	-2.214 to 2.161	0.9746		
ad	5*H*, DIV7	Normal	Unpaired parametric two-tailed *t* test	-29.84 to 11.27	0.2780		
ae	5*I*, DIV4	Normal	Unpaired parametric two-tailed *t* test	-0.2869 to 0.08355	0.2022		
af	5*I*, DIV7	Normal	Unpaired parametric two-tailed *t* test	-0.1067 to 0.1267	0.8237		
ag	6*D*, DIV12	Normal	Unpaired parametric two-tailed *t* test	-0.357 to 0.246	0.6362		
ah	6*D*, DIV14	Normal	Unpaired parametric two-tailed *t* test	-0.3671 to 0.08601	0.1601		
ai	6*D*, DIV16	Normal	Unpaired parametric two-tailed *t* test	-0.3259 to 0.2687	0.8026		
aj	6*E*, DIV12	Normal	Unpaired parametric two-tailed *t* test	-0.2159 to 0.4635	0.3689		
ak	6*E*, DIV14	Normal	Unpaired parametric two-tailed *t* test	-1.22 to 0.871	0.6674		
al	6*E*, DIV16	Normal	Unpaired parametric two-tailed *t* test	-1.186 to 2.116	0.4776		
am	6*F*	Normal	Unpaired parametric two-tailed *t* test	-0.001536 to 0.1601	0.0537		
an	7*A*	Normal	Unpaired parametric two-tailed *t* test	-2.46 to 5.546	0.4326	*U*	
ao	7*D*	Nongaussian	Mann–Whitney *U* test	-13.7 to 17.23 (median)	0.4120	29	
ap	7*E*	Nongaussian	Mann–Whitney *U* test	-0.02334 to 0.1633 (median)	0.2350	22.5	
aq	7*F*	Nongaussian	Mann–Whitney *U* test	-0.2073 to 0.6074 (median)	0.5362	28	
ar	7*I* (glutamate)	Normal	Unpaired parametric two-tailed *t* test	-0.2091 to 0.3324	0.6538		
as	7*I* (glycine)	Normal	Unpaired parametric two-tailed *t* test	-0.1241 to 0.1893	0.6822		
at	7*I* (KCl)	Normal	Unpaired parametric two-tailed *t* test	-0.0722 to 0.5019	0.1416		
au	7*I* (NMDA)	Normal	Unpaired parametric two-tailed *t* test	-0.1396 to 0.1386	0.9943	** **	** **
av	7*J* (basal)	Normal	Unpaired parametric two-tailed *t* test	-0.009 to 0.008	0.9194		
aw	7*J* (stim)	Normal	Unpaired parametric two-tailed *t* test	-0.219 to 0.024	0.8919		
ax	7*J* (post)	Normal	Unpaired parametric two-tailed *t* test	-0.018 to 0.017	0.9343		
ay	7*K*	Normal	Unpaired parametric two-tailed *t* test	-0.1198 to 0.0239	0.1375		
						*r*	*R* ^2^
az	8*A*	Normal	Pearson’s correlation	-0.5771 to 0.0044	0.0535	-0.3157	0.0997
				** **		*n*	adj *p* value
ba	8*B*	Normal	Unpaired parametric *t* test, multiple comparisons using Holm–Sidak	N/A	0.0060 -0.9991	18 genes	0.1026 -0.9999

bb	8*C*, DIV4	Normal	Unpaired parametric two-tailed *t* test	-2.717 to 2.174	0.7735		
bc	8*C*, DIV7	Normal	Unpaired parametric two-tailed *t* test	-2.219 to 3.455	0.5780		
bd	8*D*, DIV4	Normal	Unpaired parametric two-tailed *t* test	-6.521 to 7.760	0.8215		
be	8*D*, DIV7	Normal	Unpaired parametric two-tailed *t* test	-14.37 to 17.44	0.8017		
bf	8*E*, DIV4	Normal	Unpaired parametric two-tailed *t* test	-61.79 to 156.9	0.2936		
bg	8*E*, DIV7	Normal	Unpaired parametric two-tailed *t* test	-137.4 to 208.1	0.6001	*F*	adj *p* value
bh	8*H*	Normal	One-way ANOVA, Tukey’s *post hoc*	-3.239 to 7.774	0.5623	(2,29) = 0.5873	0.5722
						*H*	
bi	8*I*	Nongaussian	Kruskal–Wallis test, Dunn’s *post hoc*	N/A	0.6433	0.8824	0.9999
bj	8*J*	Nongaussian	Kruskal–Wallis test, Dunn’s *post hoc*	N/A	0.0094	9.332	0.0071
bk	8*K*	Nongaussian	Kruskal–Wallis test, Dunn’s *post hoc*	N/A	0.0070	9.927	0.0343
						*n*	adj *p* value
bl	9*C*	Normal	Unpaired parametric *t* test, multiple comparisons using Holm–Sidak	N/A	0.0057 -0.9251	18 genes	0.0976 -0.9997
bm	9*E*	Normal	Unpaired parametric two-tailed *t* test	-0.1507 to 0.4369	0.2783		
bn	9*F*	Normal	Unpaired parametric two-tailed *t* test	-5.375 to 0.6141	0.0997		
bo	9*G*	Normal	Unpaired parametric two-tailed *t* test	-48.93 to 136.5	0.2918		
bp	9*I*	Normal	Unpaired parametric two-tailed *t* test	-0.0644 to 0.5853	0.0943		
bq	9*J*	Normal	Unpaired parametric two-tailed *t* test	-0.0166 to 0.1254	0.1297		

## Results

**Figure 1.**
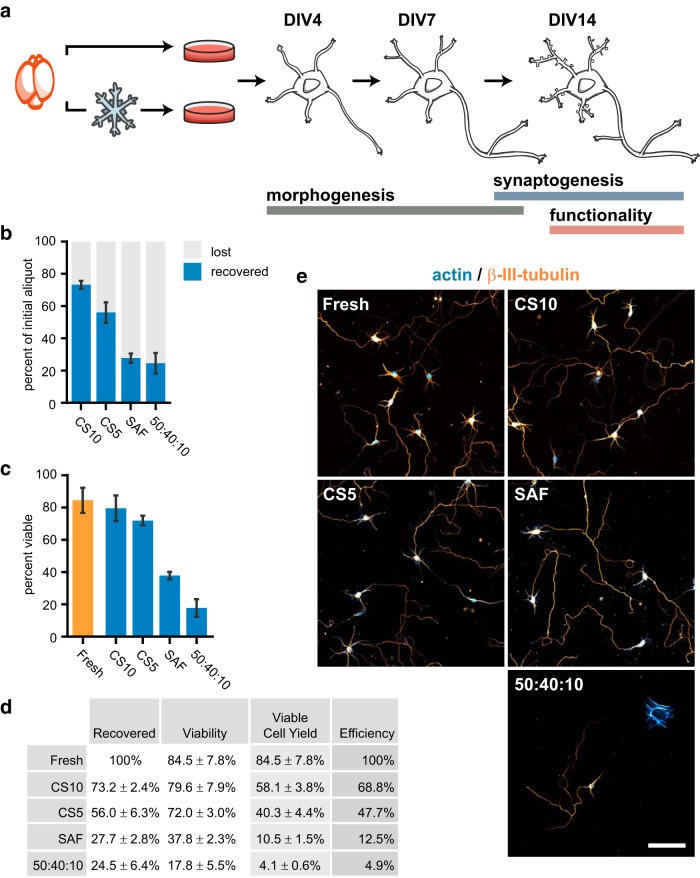
Survey of cryopreservation media for the storage of primary cortical neurons. ***A***, Experimental design and timeline for validation of cryostored neuron performance. Neurons were concurrently plated from a fresh dissection or cryostored aliquot and evaluated on indicated DIV. ***B***, Percentage of recovered and lost cells after cryostorage in test media CS10, CS5, SAF, or 50% FBS-40% DMEM-10% DMSO (50:40:10). Percentage of “recovered” cells was calculated with ((# cells alive + # cells dead) ÷ (# cells in initial aliquot)) × 100. Percentage of “lost” cells was calculated with 100% recovered. Mean ± SD, *N* = 3 source dissections. ***C***, Post-thaw viability of cells cryostored in test media compared to the viability of freshly dissected cells as evaluated by Trypan blue exclusion. Mean ± SD, *N* = 3 source dissections. ***D***, Table displaying summarized data from ***A***, ***B*** (white columns) and calculated data (gray columns). Viable cell yield for each experiment was calculated by (percentage recovered × percentage viable) ÷ 100. Efficiency was determined by normalizing viable cell yield to the average viability of a fresh dissection. ***E***, Immunofluorescence labeling of neuron-specific β-III-tubulin (orange) and fluorescently-conjugated phalloidin labeling of the actin cytoskeleton (blue) of DIV7 cells cryostored in test media. Scale bar = 100 μm.

To replace freshly dissected neurons as a model system, neurons recovered from cryostorage must be their morphologic, developmental, and functional equivalent. We designed a comprehensive validation strategy examining the properties of cultured cortical neurons after recovery from cryostorage, in comparison to freshly dissected neurons. Our experimental timeline spanned developmental stages relevant for neurobiology research, including neuronal polarization occurring by DIV4, early arborization (DIV7), initiation of synaptogenesis, and dendritic spine maturation (DIV12–DIV16; Fig. 1*A*). In addition, we performed functional assays on DIV12–DIV14 when neurons are responsive to pharmacological manipulations.

### Optimal freezing media for neuron cryopreservation identified by viability screen

To evaluate the performance of different freezing media for the cryopreservation of primary neurons, we dissociated cells from embryonic day (E)17 mouse cortices as previously described (Kaech and Banker, 2006), and resuspended aliquots in different test media. These included CS5 and CS10 (BioLife Solutions), SAF (Thermo Fisher), as well as a common traditional cryopreservation media formulation consisting of 50% FBS, 40% DMEM, and 10% DMSO (50:40:10). Aliquots were placed in a cryogenic freezing container to ensure gradual cooling, were initially stored in a -80°C freezer, and then transferred after 2 d to liquid nitrogen for long-term storage.

Within a week of cryostorage, cell aliquots were thawed and resuspended in neuronal plating media, and their post-thaw recovery and viability were immediately assessed via Trypan blue exclusion assay. The total number of recovered cells (live and dead combined) was consistently lower than the number of cells initially aliquoted across all examined media (Fig. 1*B*). This suggests damage to, and subsequent loss of, cells during the processes of freezing, thawing, or removal of DMSO, consistent with previous reports (Higgins et al., 2011). CS10 provided the highest cell recovery with an average of only 27% cell loss, compared to CS5 (44%), SAF (72%), and 50:40:10 (75%). Cells stored in CS10 and CS5 each achieved 70% post-thaw cell viability or greater (Fig. 1*C*). In contrast, cells preserved in SAF media achieved an average viability of 38%, while 50:40:10 exhibited poor viability, with an average of 18% viable cells on thawing.

Viable cell yield, a metric which accounts for both viability and loss, was calculated for each test media. This metric was normalized to the viable cell yield of a fresh dissection to evaluate the relative efficiency of cryopreservation (Fig. 1*D*). CS10 permitted the recovery of viable cells with 68.8% efficiency. CS5 performed at 47.7% efficiency, while SAF and 50:40:10 each provided <15% efficiency. In all examined media and conditions, cells developed into glia or neurons (Fig. 1*E*). Together, these results demonstrate that viable primary neural cells can be reliably recovered after freezing using commercially-available media and the best-performing media with respect to viability and recovery on thawing was CS10.

### Neurons are more sensitive to cryostorage than glia

**Figure 2.**
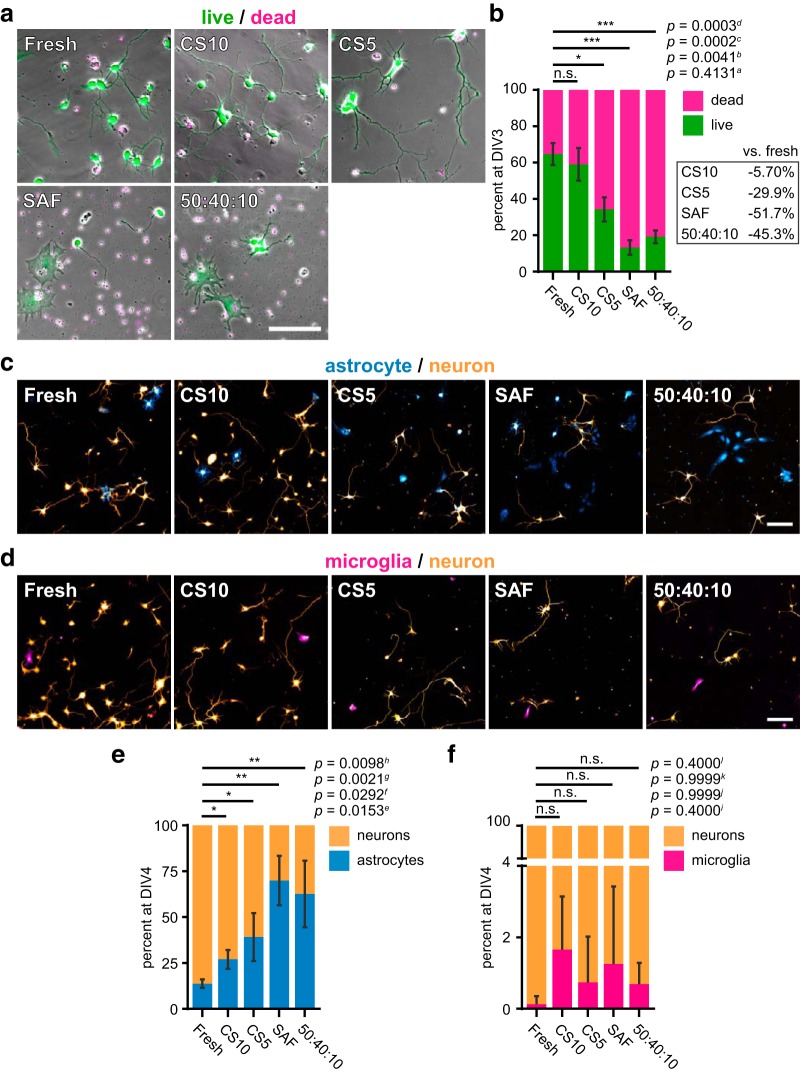
Long-term survival of neurons cryostored in CS10 only slightly lower than a fresh dissection. ***A***, Cell survival after cryostorage and recovery in test media compared to freshly dissected cells at DIV3. Live cells were labeled by calcein (green), and dead cells are indicated by ethidium homodimer labeling (magenta). Scale bar = 100 μm. ***B***, Quantification of live/dead assay at DIV3. Table displays average change in percentage survival of cryostored cells versus fresh cells. Mean ± SD, *N* = 3 source dissections, *n* > 2300 cells per condition. ***C***, ***D***, Immunofluorescence labeling of fresh or cryostored cells fixed at DIV4. Cells were labeled with antibodies against the neuron-specific marker β-III-tubulin (***C***, ***D***; orange), astrocyte marker S100 (***C***; blue), or microglia marker Iba1 (***D***; magenta). Scale bar = 100 μm. ***E***, Percentage of cell population at DIV4 labeled with β-III-tubulin or S100. Mean ± SD, *N* = 3 source dissections, *n* > 1250 cells per condition. ***F***, Percentage of cell population at DIV4 labeled with β-III-tubulin or Iba1. Mean ± SD, *N* = 3 source dissections, *n* > 250 cells per condition. All statistical comparisons made by unpaired parametric *t* test (***B***, ***E***) or Mann–Whitney *U* test (***F***). n.s. = not significant, *p < 0.05, **p < 0.01, ***p < 0.001.

Cell death pathways, including apoptotic and necrotic pathways, are activated by cryodamage (Baust et al., 2002). Therefore, an effect of cryostorage on survival may not be apparent until hours or days after thawing. To assess the long-term survival of cryostored cells, we performed a fluorescence-based survival assay, in which intact cells are labeled with calcein AM and dying cells with ethidium homodimer-1 (Fig. 2*A*). Compared to time-matched cultures from a fresh dissection, cell survival at 3 d postplating was significantly lower in cultures recovered from cryostorage in CS5, SAF, and 50:40:10, while the percentage of live CS10-cryostored cells was only 5.7% lower than that observed in freshly dissected cultures (Fig. 2*B*).

Dissociated primary cultures from embryonic cortex contain a mixed population of cells (Miller and Gauthier, 2007). To examine the long-term survival of neurons compared to glial cells, we performed immunofluorescence labeling on fresh and cryostored cultures, using antibodies against both neuron-specific β-III-tubulin and various glial subtype markers, including S100 for astrocytes, Iba1 for microglia, or Olig2 for oligodendrocytes (Lyck et al., 2008; Fig. 2*C*,*D*). Cultured cells were fixed at DIV4, when neurons and glial cells can be easily distinguished. S100-positive astrocytes were the most commonly identified non-neuronal cell type under both conditions (Fig. 2*C*,*E*). Iba1-positive microglia represented only a very small fraction of cells (Fig. 2*D*,*F*), while oligodendrocytes were not detected, consistent with these cells being relatively few in embryonic cortex (data not shown; Miller and Gauthier, 2007). In all cryopreserved cultures, the ratio of astrocytes to neurons was significantly higher compared to fresh cultures (Fig. 2*E*), suggesting that neurons are likely more sensitive to cryostorage than glial cells. To prevent glia overproliferation, we routinely add the mitotic inhibitor AraC to all cultures at DIV4.

Together, these results demonstrate that of all examined media, CS10 is optimal for the cryopreservation of primary neural cells, providing the highest recovery, post-thaw viability, and cell survival. As these gains in efficiency are substantial improvements over prior methodologies (see Discussion), CS10 holds the most promise of everyday utility, and was hence made the focus of our validation studies.

### Expression levels of neurodevelopmental genes are normal in cryostored samples

**Figure 3.**
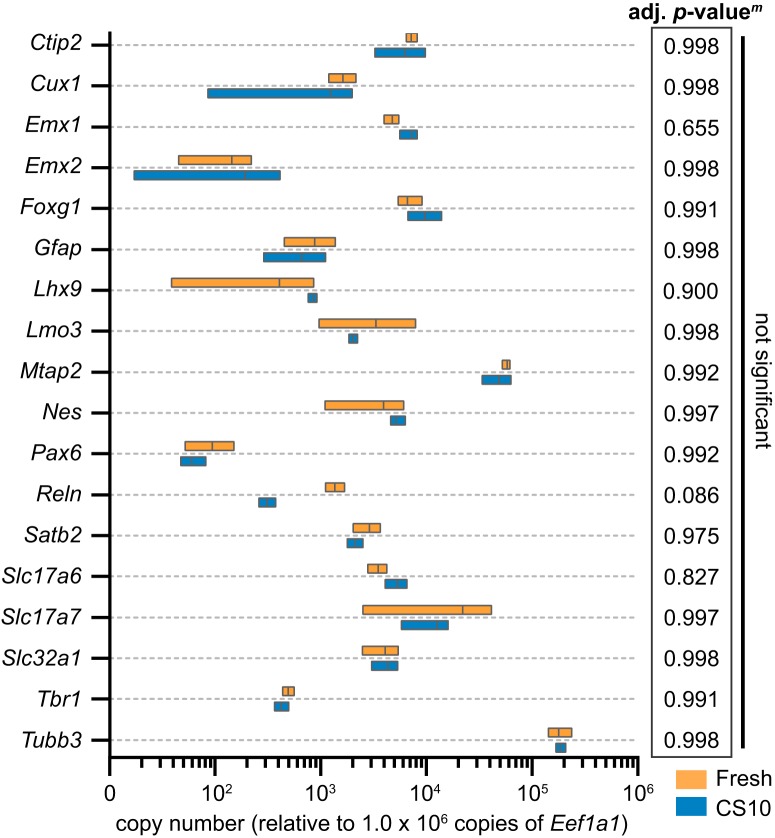
Expression of key neurodevelopmental genes is unchanged following recovery from cryostorage. RT-qPCR of RNA samples from fresh or CS10-cryostored cortical cultures collected at DIV12. C_T_s were normalized to housekeeping genes and relative copy numbers were generated using 2^-ΔCT^ × 10^6^. Floating bar graph spans the minimum and maximum data points, vertical line denotes mean. *N* = 3 source dissections. All statistical comparisons made by unpaired parametric *t* test with Holm–Sidak correction for multiple comparisons. Multiplicity adjusted *p* value for each comparison listed in table (right).

At the time of dissection, cortical and hippocampal neurogenesis is robustly underway, and neurons are just beginning to terminally differentiate (Miller and Gauthier, 2007). to determine whether cryostorage influences development and maturation compared to freshly dissected cells, we examined the expression of several key neurodevelopmental markers used to confirm appropriate differentiation of cortical neurons from embryonic stem cells (Gaspard et al., 2009; Terrigno et al., 2018). These include *Nes*, *Pax6* (neural progenitor cells), *Emx1*, *Emx2*, *Foxg1* (cells derived from dorsal telencephalon), *Gfap* (astrocytes), *Mtap2* (MAP2) and *Tubb3* (β-III-tubulin, terminal differentiation), *Tbr1*, *Satb2*, *Ctip2* (cortical neurons), *Cux1*, *Lmo3*, *Reln* (cortical subtypes), *Lhx9* (hippocampal lineage), *Slc17a7* (VGLUT1), *Slc17a6* (VGLUT2), and *Slc32a1* (VGAT, neurotransmitter transporters). RT-qPCR of RNA samples collected at DIV12 indicated no significant differences in gene expression by *t* test with Holm–Sidak correction for multiple comparisons, used to reduce Type I statistical error (Fig. 3). However, expression of *Reln* was consistently lower in samples recovered from cryostorage with an average fold change of 0.23 ± 0.03 compared to freshly dissected samples, although this did not reach statistical significance (multiplicity adjusted *p* = 0.086). These data suggest that overall, CS10-cryostored samples are developmentally similar to their freshly-obtained counterparts.

### Outgrowth and arborization of neuronal processes are comparable between fresh and cryostored neurons

**Figure 4.**
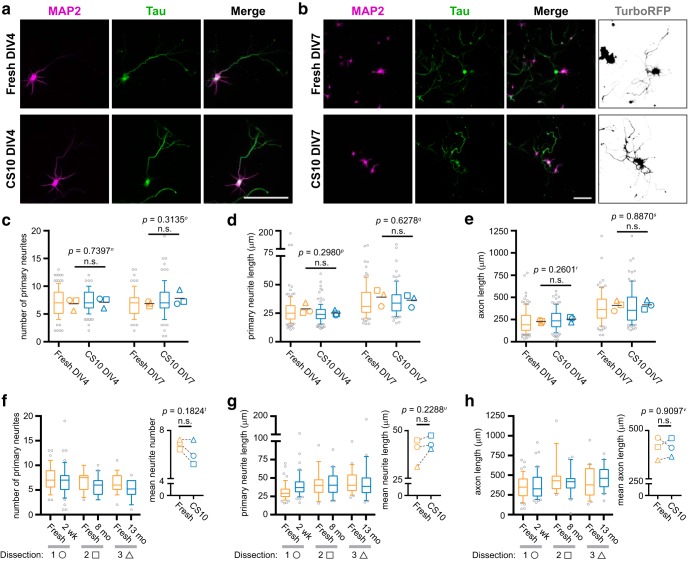
Cryostorage does not alter neuron morphogenesis. ***A***, ***B***, Immunofluorescence labeling of freshly dissected or CS10-cryostored cells, using antibodies against the dendrite marker MAP2 (magenta) and axon marker Tau (green). Cells were fixed on DIV4 (***A***) or DIV7 (***B***). Cultures grown to DIV7 were also infected with a sub-saturating titer of cytosolic TurboRFP-encoding lentivirus to aid in tracing (inverted white). Scale bar = 100 μm. ***C*–*E***, Quantification of number of primary neurites (***C***), primary neurite length (***D***), and axon length (***E***) of traced cortical neurons at indicated timepoints and conditions. Box-and-whisker plots show data pooled from three independent experiments; whiskers indicate 10th to 90th percentile, box indicates 1st to 3rd quartile, center line is median, gray points are data outside 10th to 90th percentile. Symbols (○◻△) denote the mean value calculated for each biological replicate, horizontal line is the grand mean. *N* = 3 source dissections, *n* > 90 neurons per condition and time point. Statistical comparisons between biological replicate means made by unpaired parametric *t* test. ***F–H***, Quantification of number of primary neurites (***F***), primary neurite length (***G***), and axon length (***H***) of freshly dissected cortical neurons compared to aliquots from the same source dissection cryostored for indicated durations, and traced at DIV7. Box-and-whisker plots show data from each experiment. *n* > 14 neurons per condition and time point. Inset graph plots mean for each dataset, symbols (○◻△) denote each source dissection, dashed line connects fresh-CS10 matched pairs. Statistical comparisons between matched means made by paired parametric *t* test. n.s. = not significant. Fresh DIV7 morphometric data for each dissection were extracted from ***C–E***.

Cortical neurons undergo dramatic morphologic changes in the first week of culture. We performed morphometric analysis of neurons on DIV4, shortly after axon specification, and on DIV7, when elaboration of the dendrites and axonal outgrowth occurs (Barnes and Polleux, 2009). Cells were fixed and immunolabeled for Tau and MAP2, demarcating axons and dendrites, respectively (Fig. 4*A*,*B*). A TurboRFP lentivirus was also used to sparsely label cells thereby facilitating tracing of individual neurons at DIV7 when neuronal arbors are complex. Comparison of fresh and CS10-cryostored neurons revealed that the number of primary neurites (Fig. 4*C*), their length (Fig. 4*D*), as well as axon length (Fig. 4*E*) were not significantly different between the two conditions.

Using cryopreservation, cells obtained by fresh dissection can be stored and recovered at a later time, thereby permitting examination of morphogenesis before and after cryostorage within the same sample. Morphometric data from “fresh DIV7” (Fig. 4*C–E*) were split into their three constituent source dissections, and a frozen aliquot from each of those dissections was thawed after the indicated duration in cryostorage. At DIV7, there were no significant changes in neurite number (Fig. 4*F*), neurite length (Fig. 4*G*), or axon length (Fig. 4*H*) as determined by paired *t* test on the means derived from each experiment, indicating that cryostorage itself is not sufficient to impact neuronal morphogenesis.

**Figure 5.**
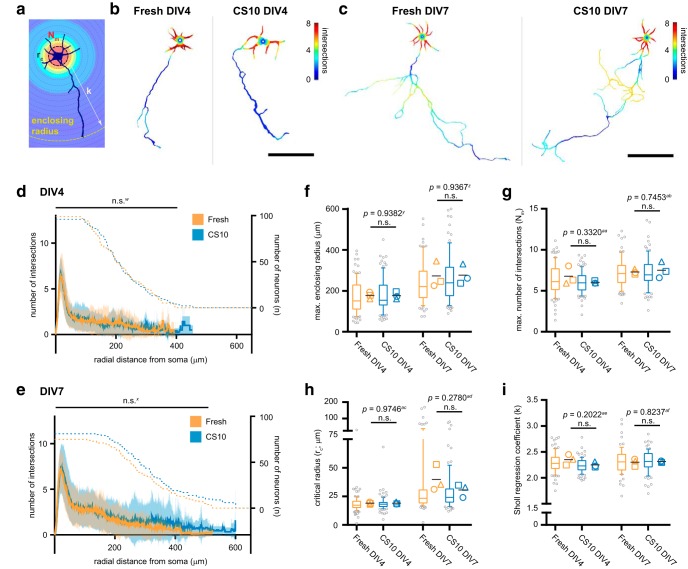
Neuron arborization and branch complexity are unaffected by cryostorage. ***A***, Diagram of Sholl analysis with indicated metrics: enclosing radius, maximum number of intersections (N_m_), critical radius (r_c_), and Sholl regression coefficient (k). ***B***, ***C***, Representative masks of neuron tracings illustrate number of intersections (spectrum scale bar) detected by Sholl analysis of DIV4 (***B***) and DIV7 (***C***) cortical neurons from indicated conditions. Scale bar = 100 μm. ***D***, ***E***, Sholl profiles of freshly dissected (orange) or CS10-cryostored (blue) neurons at DIV4 (***D***) and DIV7 (***E***). Data are represented as mean (solid line, left *y*-axis) ± SD (shading); number of neurons (*n*; dashed line, right *y*-axis). ***F–I***, Quantification of enclosing radius (***F***), N_m_ (***G***), r_c_ (***H***), and *k* (***I***) of Sholl-analyzed tracings at indicated timepoints and conditions. Box-and-whisker plots show data pooled from three independent experiments; whiskers indicate 10th to 90th percentile, box indicates 1st to 3rd quartile, center line is median, gray points are data outside 10th to 90th percentile. Symbols (○◻△) denote the mean value calculated for each biological replicate, horizontal line is the grand mean. *N* = 3 source dissections, *n* > 90 neurons per condition and time point. Statistical comparison between mean Sholl profiles at each radius made by unpaired parametric *t* test with Holm–Sidak correction for multiple comparisons (***D***, ***E***), and statistical comparison between biological replicate means for Sholl metrics made by unpaired parametric *t* test (***F–I***). n.s. = not significant.

To quantitate branch complexity, we performed Sholl analysis (Sholl, 1953). In this analysis, concentric circles emanate radially from the cell soma, and intersect neuronal processes as their radius expands. The number of intersections at each radius provides a profile of neuronal arborization, which is described by metrics including the maximum enclosing radius, maximum number of intersections (N_m_), the critical radius (r_c_; radius at which N_m_ occurs) and the Sholl regression coefficient (k; rate of decay of branch density with distance from the soma; Fig. 5*A*; Sholl, 1953; Ristanović et al., 2006). These metrics quantitate the relationship between branch density and the expanse of the neuritic field, and estimate the morphologic complexity of a neuron. Representative neuronal tracings at DIV4 and DIV7 illustrate the Sholl method with a heatmap of intersection density (Fig. 5*B*,*C*).

Sholl profiles and metrics for neurons traced in Figure 4 were individually determined, and then averaged into a mean Sholl profile for each time point and condition. At DIV4 and DIV7, the mean Sholl profile of freshly dissected and CS10-cryostored neurons were comparable at each radius-step from the soma (Fig. 5*D*,*E*). The processes of cryostored neurons extended further distances from the soma than freshly dissected neurons at both timepoints, although this difference was attributable to only a few neurons (DIV4 *n* = 2, DIV7 *n* = 5) and was not statistically significant (Fig. 5*F*). The metrics N_m_, r_c_, and *k* were indistinguishable between conditions at each time point examined (Fig. 5*G–I*). Together, these results suggest that critical aspects of neuronal morphogenesis, including neurite outgrowth, axon elongation, and branch elaboration are unperturbed by CS10-mediated cryostorage.

### Synaptogenesis occurs normally in cryopreserved neurons

**Figure 6.**
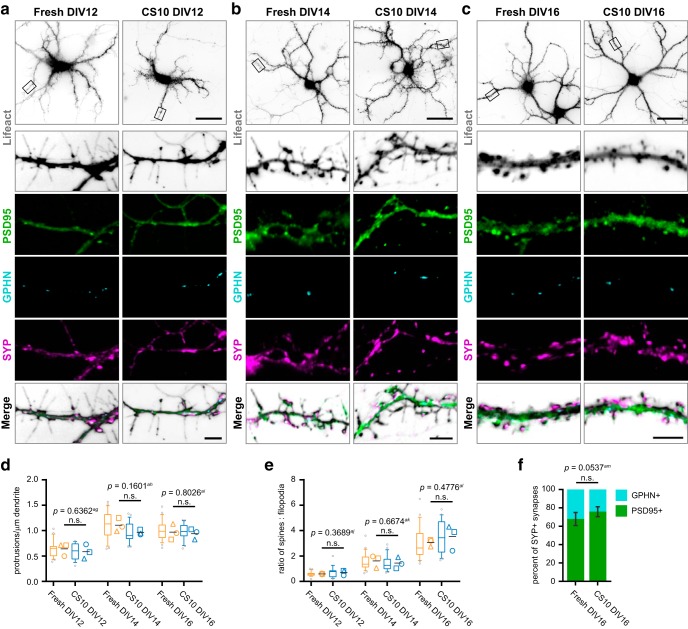
Synaptogenesis occurs normally in neurons following cryostorage and recovery. ***A–C***, Neurons expressing Lifeact-mRuby2 (inverted white) from indicated conditions were fixed at DIV12 (***A***), DIV14 (***B***), and DIV16 (***C***), and immunolabeled using antibodies against excitatory postsynaptic marker PSD95 (NeuroMab; green), inhibitory postsynaptic marker gephyrin (GPHN; cyan), and presynaptic marker synaptophysin (SYP; magenta). Bottom panel is magnified segment of boxed dendrite region. Scale bar = 100 μm (top) and 5 μm (bottom panel). ***D***, Density of all actin-rich protrusions from secondary dendrites at indicated timepoints and conditions. ***E***, Ratio of dendritic spines to dendritic filopodia as determined by presence or absence of PSD95 on actin-rich protrusions at indicated timepoints and conditions. Box-and-whisker plots (***D***, ***E***) show data pooled from three independent experiments; whiskers indicate 10th to 90th percentile, box indicates 1st to 3rd quartile, center line is median, gray points are data outside 10th to 90th percentile. Symbols (○◻△) denote the mean value calculated for each biological replicate, horizontal line is the grand mean. ***F***, Percentage of PSD95-positive excitatory synapses and GPHN-positive inhibitory synapses at DIV16 from indicated conditions. Mean ± SD, *N* = 3 source dissections, *n* = 6–7 neurons per condition. All statistical comparisons between biological replicate means made by unpaired parametric *t* test. n.s. = not significant.

At around two weeks in culture, dendrites extend filopodia-like protrusions, which can form nascent contacts that later remodel into mature synapses (Yuste and Bonhoeffer, 2004). To determine whether cryopreservation affects filopodial outgrowth or subsequent synaptogenesis, we examined dendritic protrusion density and synapse maturity during the respective period. Neurons expressed Lifeact-mRuby2 to reveal actin-rich dendritic filopodia and spines. We assessed the prevalence of excitatory and inhibitory synapses using immunofluorescence labeling for the excitatory postsynaptic marker PSD95, the inhibitory postsynaptic marker gephyrin (GPHN), and the presynaptic marker synaptophysin (SYP; Micheva et al., 2010; Fig. 6*A–C*). The total density of protrusions was comparable between fresh and CS10-cryostored neurons at DIV12, DIV14, and DIV16 (Fig. 6*D*). The ratio of dendritic spines to filopodia increased with maturation as expected and was not significantly different between the two conditions at each time point examined (Fig. 6*E*). Quantification of PSD95- and GPHN-positive synapses revealed a trend toward increased prevalence of excitatory over inhibitory synapses in cryopreserved neurons at DIV16; however, this fell short of statistical significance (Fig. 6*F*). Hence, CS10-mediated cryostorage has no significant impact on the course of synaptogenesis, nor does it substantially affect the density of synapses or type of synapses formed.

### Cryostored neurons are functionally similar to neurons obtained from fresh dissection

**Figure 7.**
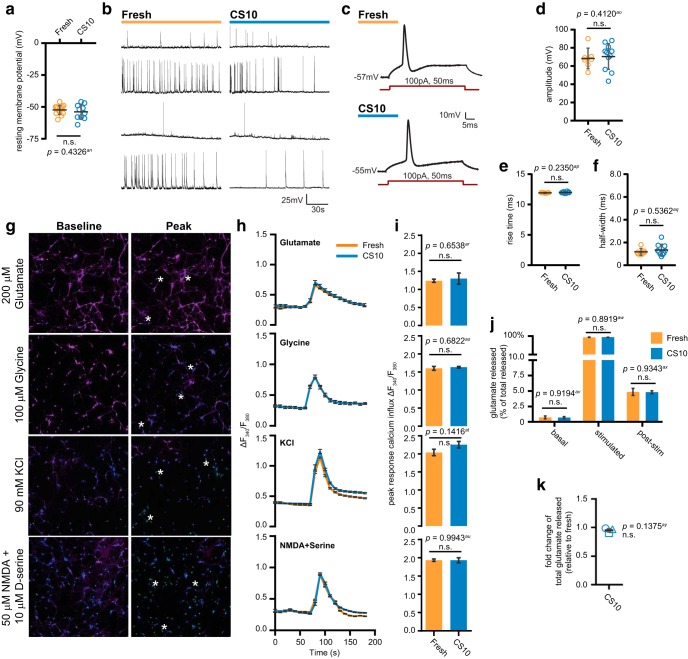
Cryopreserved cortical neurons have normal spontaneous and stimulated activity. ***A***, Resting membrane potential of fresh and CS10-cryostored neurons recorded by whole-cell patch clamp. Scatterplot of data points pooled from one to two biological replicates. Mean ± SD, *N* = 1–2 source dissections, *n* = 10–14 neurons per condition. ***B***, Examples of whole-cell patch clamp recordings of neurons exhibiting spontaneous activity from fresh or CS10-cryostored neurons at DIV12–DIV14. ***C***, Representative tracings of evoked APs after 100-pA current injection for 50 ms (red). ***D–F***, Quantification of evoked AP parameters including amplitude (***D***), rise time (***E***), and half-width (***F***). Scatterplot of data points pooled from one to two biological replicates. Mean ± SD, *N* = 1–2 source dissections, *n* = 7–11 neurons per condition. ***G***, Fluorescent images of calcium imaging experiments showing the representative response of CS10-cryostored neurons to the indicated trigger at DIV14. Baseline (left column) Fura-2 fluorescence F_340_/F_380_ ratio, and peak (right column) Fura-2 fluorescence F_340_/F_380_ ratio, in response to 15-s stimulation with the indicated trigger. Scale bar = 100 μm. ***H***, Traces of mean change in Fura-2 fluorescence F_340_/F_380_ ratio over time for the indicated triggers and conditions. ***I***, Summary bar graphs showing the peak fluorescence response (as variation from baseline, adjusted for background) for the indicated triggers. Mean ± SEM, *N* = 1 source dissection, *n* > 75 neurons per condition. ***J***, Glutamate release by DIV14 neurons from indicated conditions in non-depolarizing solution (basal), after 15 min of stimulation with KCl (stimulated), and after return to non-depolarizing solution (post-stim). Glutamate release at each time point is presented as a percentage of the total glutamate released. ***K***, Fold change of total glutamate released by CS10-cryostored neurons relative to fresh. Mean ± SEM, *N* = 3 source dissections. Statistical comparisons made by unpaired parametric *t* test (***A***, ***I–K***) or Mann–Whitney *U* test (***D–F***). n.s. = not significant.

To complete our assessment of cryostored neurons, we sought to determine whether neurons recovered from cryopreservation establish functional neuronal networks by measuring spontaneous electrical activity and stimulus-evoked responses. We used whole-cell patch clamp electrophysiology to record fresh and CS10-cryostored neurons cultured to DIV12–DIV14. Recovery from cryostorage in CS10 did not perturb the resting membrane potential of neurons (Fig. 7*A*). Neurons cultured under either condition exhibited a diverse array of spontaneous firing patterns (Fig. 7*B*), which reflected the diversity typical of cortical neurons (Connors et al., 1982; McCormick et al., 1985; Connors and Gutnick, 1990). This suggests that cryostored neurons form complex functional networks similar to that of freshly cultured neurons. Due to the inherent heterogeneity of their spontaneous behavior, we analyzed the wave form parameters of evoked APs elicited by current injection (Fig. 7*C*) and quantified peak amplitude (Fig. 7*D*), rise time (Fig. 7*E*), and half-width (Fig. 7*F*). No significant differences were detected between fresh and CS10-cryostored neurons for any metric, demonstrating that following cryostorage, neurons are capable of similar electrophysiological behaviors to those obtained from fresh dissection.

To test whether cryostorage affects ligand- and voltage-gated Ca^2+^ channel function, we used a ratiometric calcium imaging approach using Fura-2 AM. We tested neurons for their ability to elicit calcium influx in response to the neurotransmitters glutamate (targeting ionotropic and metabotropic glutamate receptors), glycine (glycine receptor) or NMDA + D-serine (NMDA receptor). Voltage-gated Ca^2+^ channels were activated by depolarization using 90 mM KCl (Fig. 7*G*). Ca^2+^ influx elicited by each trigger was measured by determining the change in fluorescence F_340_ over F_380_ (Fig. 7*H*). No statistical differences were found between fresh and CS10-cryostored cultures for the peak Ca^2+^ influx elicited by any of the examined stimuli (Fig. 7*I*).

Lastly, we measured the ability of cryopreserved neurons to release glutamate in response to sustained depolarization. Freshly dissected and CS10-cryostored cultures were stimulated with 90 mM KCl to induce depolarization-dependent glutamate release. Media samples were collected before, during, and after stimulation (Fig. 7*J*). Both populations released equal proportions of their glutamate content in response to stimulation (Fig. 7*J*). The relative amount of glutamate released by CS10-cryostored cultures was also not significantly different from fresh cultures (Fig. 7*K*, mean fold change ± SD = 0.952 ± 0.04). Together, these results demonstrate that the functional properties of cryopreserved cortical neurons are comparable to freshly dissected neurons.

### Cortical neurons recovered from extended cryostorage have greater performance heterogeneity than neurons recovered from shorter-term storage

**Figure 8.**
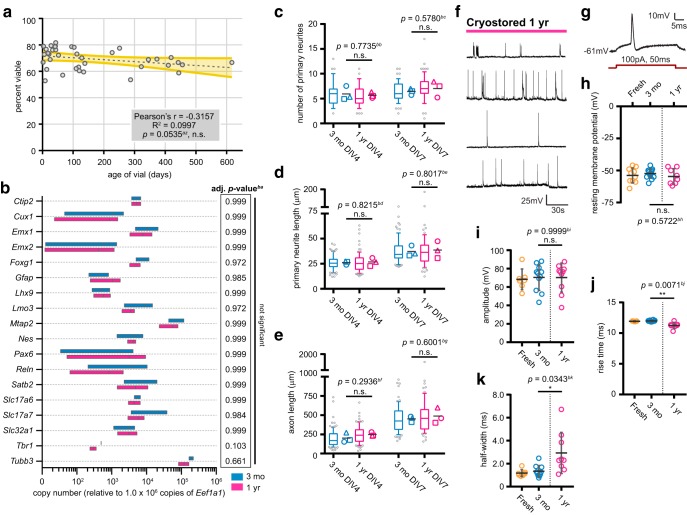
Duration of cryostorage does not impact several essential neuronal properties but may affect electrophysiological performance. ***A***, Viability of cells cryostored in CS10 media and recovered at various times after freezing. Dashed gray line = linear regression; yellow shaded area = 95% confidence band. *N* = 12 source dissections, *n* = 38 cryostored aliquots. Statistical analysis performed by Pearson’s correlation test. n.s. = not significant. ***B***, RT-qPCR of RNA samples from DIV12 cortical cultures cryostored for three months or one year. C_T_s were normalized to housekeeping genes and relative copy numbers were generated using 2^-ΔCT^ × 10^6^. Floating bar graph spans the minimum and maximum data points, vertical line denotes mean. *N* = 2 source dissections, *n* = 3 thawed aliquots. Statistical comparisons made by unpaired parametric *t* test with Holm–Sidak correction for multiple comparisons. Multiplicity adjusted *p* value for each comparison listed in table (right). ***C–E***, Quantification of number of primary neurites (***C***), primary neurite length (***D***), and axon length (***E***) of traced cortical neurons at indicated timepoints. Box-and-whisker plots show data pooled from three independent experiments; whiskers indicate 10th to 90th percentile, box indicates 1st to 3rd quartile, center line is median, gray points are data outside 10th to 90th percentile. Symbols (○◻△) denote the mean value calculated for each biological replicate, horizontal line is the grand mean. *N* = 2 source dissections, *n* > 65 neurons per condition and time point. Statistical comparisons between biological replicate means made by unpaired parametric *t* test (***C–E***). ***F***, Examples of whole-cell patch clamp recordings of spontaneous activity from neurons recovered after one-year cryostorage in CS10 at DIV12–DIV14. ***G***, Representative tracing of evoked APs after 100-pA current injection for 50 ms (red). ***H***, Resting membrane potential of neurons recovered after one-year cryostorage in CS10 recorded by whole-cell patch clamp, alongside freshly dissected and three month-cryostored neurons reproduced from Figure 7 for comparison (separated by dashed line). Scatterplot of data points pooled from one to two biological replicates. Mean ± SD, *N* = 1–2 source dissections, *n* = 8–14 neurons per condition. ***I–K***, Quantification of evoked AP parameters including amplitude (***I***), rise time (***J***), and half-width (***K***) of neurons recovered after one-year cryostorage in CS10, alongside freshly dissected and three month-cryostored neurons reproduced from Figure 7 for comparison (separated by dashed line). Scatterplot of data points pooled from one to two biological replicates. Mean ± SD, *N* = 1–2 source dissections, *n* = 7–11 neurons per condition. Statistical comparisons made by one-way ANOVA with Tukey’s test for multiple comparisons (***H***) or Kruskal–Wallis test with Dunn’s test for multiple comparisons (***I–K***). n.s. = not significant, *p < 0.05, **p < 0.01.

To further examine the utility of our method, we tested whether aliquots cryostored for various durations of time performed equivalently. Cell viability was not substantially affected by the duration of cryostorage, as cells frozen in CS10 for over a year exhibited only a small decrease in viability compared to vials stored for a few days (Fig. 8*A*).

We next examined whether cortical neurons subjected to long-term cryostorage performed equivalently to neurons stored for a shorter duration using key tests from our battery of validation assessments. We thawed aliquots cryostored for three months or one year, and compared gene expression, morphometric parameters, and electrophysiological performance. Expression of neurodevelopmental genes was similar between the two conditions at DIV12 (Fig. 8*B*). *Tbr1* levels were slightly but not significantly reduced in one-year-old compared to three-month-old samples (fold change 0.59 ± 0.08, multiplicity adjusted *p* = 0.103). Neurons from short- and long-term cryostorage had comparable primary neurites (Fig. 8*C*,*D*) and axon lengths (Fig. 8*E*), indicating that the duration of cryostorage likely does not alter morphogenesis.

Finally, we examined the electrophysiological properties of cortical neurons recovered after three months or one year of cryostorage. Recordings of spontaneous neuronal firing patterns revealed an intrinsic heterogeneity similar to our previous observations (Figs. 7*A*, 8*F* ). In addition, the resting membrane potential and peak amplitude of evoked APs were similar among fresh, three-month-, and one-year-old cryostored neurons (Fig. 8*G–I*; fresh and three-month data reproduced from Fig. 7 for comparison). However, the rise time was significantly reduced, and the half-width was significantly increased, for evoked APs from neurons cryostored for one year (Fig. 8*J*, three months vs one year *p* = 0.007; Fig. 8*K*, three months vs one year *p* = 0.034). In addition, one-year-old cryostored neurons demonstrated a larger variability in rise time and half-width of individual evoked APs [coefficient of variation (%; 100 × SD ÷ mean) for fresh, three months, one year: rise time 0.3%, 1.0%, and 4.5%; half-width 25.6%, 37.8%, and 61.0%], suggesting greater cell heterogeneity. Hence, the electrophysiological properties of neurons may be sensitive to the duration of cryostorage or storage conditions.

### CryoStor CS10 is suitable for the cryopreservation of primary embryonic hippocampal neurons

**Figure 9.**
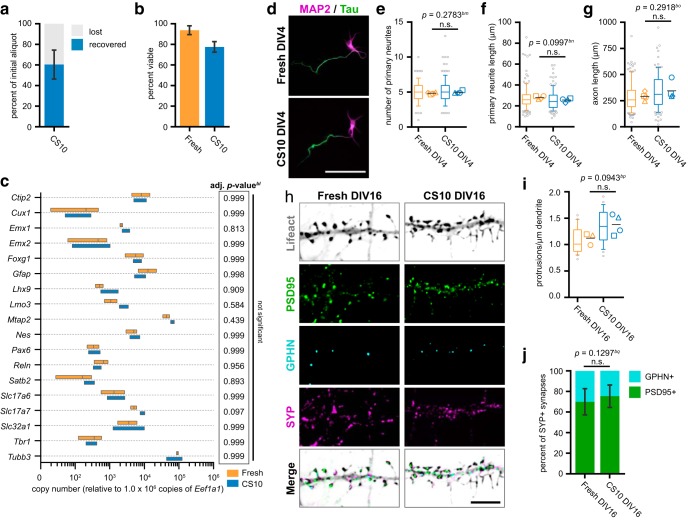
CS10 is effective for the cryostorage of primary hippocampal neurons. ***A***, Percentage of recovered and lost hippocampal cells after cryostorage in CS10. Percentage of “recovered” cells was calculated with ((# cells alive + # cells dead) ÷ (# cells in initial aliquot)) × 100. Percentage of “lost” cells was calculated with 100% recovered. Mean ± SD, *N* = 7 source dissections. ***B***, Post-thaw viability of CS10-cryostored hippocampal cells compared to the viability of freshly dissected cells as evaluated by Trypan blue exclusion. Mean ± SD, *N* = 7 source dissections. ***C***, RT-qPCR of RNA samples from fresh or CS10-cryostored hippocampal cultures collected at DIV12. C_T_s were normalized to housekeeping genes and relative copy numbers were generated using 2^-ΔCT^ × 10^6^. Floating bar graph spans the minimum and maximum data points, vertical line denotes mean. *N* = 3 source dissections. Statistical comparisons made by unpaired parametric *t* test with Holm–Sidak correction for multiple comparisons. Multiplicity adjusted *p* value for each comparison listed in table (right). ***D***, Immunofluorescence labeling of freshly dissected or CS10-cryostored hippocampal neurons at DIV4, using antibodies against the dendrite marker MAP2 (magenta) and axon marker Tau (green). Scale bar = 100 μm. ***E–G***, Quantification of number of primary neurites (***E***), primary neurite length (***F***), and axon length (***G***) of freshly dissected or CS10-cryostored hippocampal neurons at DIV4. Box-and-whisker plots show data pooled from four independent experiments; whiskers indicate 10th to 90th percentile, box indicates 1st to 3rd quartile, center line is median, gray points are data outside 10th to 90th percentile. Symbols (○◻△◇) denote the mean value calculated for each biological replicate, horizontal line is the grand mean. *N* = 4 source dissections, *n* > 115 neurons per condition. ***H***, Immunofluorescence labeling of freshly dissected or CS10-cryostored hippocampal neurons expressing Lifeact-mRuby2 (inverted white) at DIV16, using antibodies against excitatory postsynaptic marker PSD95 (Novus; green), inhibitory postsynaptic marker gephyrin (GPHN; cyan), and presynaptic marker synaptophysin (SYP; magenta). Scale bar = 5 μm. ***I***, Density of actin-rich protrusions from secondary dendrites ay DIV16 from indicated conditions. ***J***, Percentage of PSD95-positive excitatory synapses and GPHN-positive inhibitory synapses at DIV16 from indicated conditions. Mean ± SD, *N* = 4 source dissections, *n* > 16 neurons per condition. All statistical comparisons between biological replicate means made by unpaired parametric *t* test (***E–G***, ***I***, ***J***). n.s. = not significant.

To explore the versatility of our cryopreservation method, we assessed CS10’s ability to faithfully preserve another embryonic neural tissue commonly used in primary neuron culture. We harvested hippocampal neurons from E17 mouse embryos, and compared the viability and development of cryostored hippocampal neurons to freshly dissected cells. On thawing, aliquots of hippocampal cells achieved 60% recovery and 78% viability (Fig. 9*A*,*B*), giving a viable cell yield of 47.1 ± 1.2%, and an efficiency of 50.2% compared to fresh dissection.

RT-qPCR of RNA samples collected at DIV12 revealed that overall, expression of neurodevelopmental markers is unaffected by cryostorage (Fig. 9*C*). CS10-cryostored samples expressed slightly higher levels of *Slc17a7* (VGLUT1, average fold change 1.86 ± 0.13, multiplicity adjusted *p* = 0.097), as well as slightly higher *Mtap2* (MAP2, average fold change 1.48 ± 0.1, multiplicity adjusted *p* = 0.439), although this did not reach statistical significance. Fresh and CS10-cryostored hippocampal neurons were fixed and immunolabeled for dendrite and axonal markers on DIV4 (Fig. 9*D*) and traced for morphometric analysis. No significant differences in neurite number, neurite length, or axon length (Fig. 9*E–G*) were detected between the two conditions. Finally, we assessed synaptogenesis by performing immunofluorescence labeling of synapse markers on Lifeact-expressing fresh and cryostored hippocampal neurons fixed at DIV16 (Fig. 9*H*). The density of synapses and filopodia was slightly increased in cryostored cells; however, this difference did not reach statistical significance (Fig. 9*I*). Further, the prevalence of excitatory and inhibitory synapses as indicated by PSD95 or gephyrin immunopositivity, respectively, was unchanged in hippocampal neurons recovered from cryostorage (Fig. 9*J*). Thus, CS10 provides effective cryoprotection for a variety of embryonic neural tissues, yielding neurons that are comparable to those obtained by fresh dissection.

## Discussion

A successful cryopreservation methodology must achieve two main objectives: (1) high recovery rate to meet experimental demands, and (2) preservation of physiologic properties of the recovered cells. The inability to reliably recover sufficiently high yields of healthy primary neurons from cryostorage using traditional freezing media has discouraged the adoption of cryopreservation in everyday cellular neurobiology research. Here, we demonstrate that cryopreservation of dissociated primary cortical and hippocampal neurons from mice is not only feasible and efficient but yields cells that are, by our methods, equivalent to freshly dissected neurons.

We screened several freezing media and identified CryoStor CS10, a commercially-available reagent that provided efficient recovery and robust survival of healthy cortical and hippocampal neurons from cryostorage. Using a comprehensive validation strategy, we assessed gene expression, neuronal morphogenesis and synaptogenesis at critical developmental timepoints, as well as the functional response of neurons to various stimuli. We found that cryopreservation in CS10 media did not compromise any of the examined fundamental neuronal properties.

### CS10 substantially improves neuron yield and long-term survival compared to other cryopreservation reagents

CryoStor is part of a new generation of freezing media formulated to mimic intracellular composition, and thereby mitigate the osmotic stress experienced by the cell during cryopreservation. CryoStor incorporates sugars, salts, buffering agents, antioxidants, and both cell-permeating and non-permeating cryoprotectants (Hawkins et al., 2017). These additives provide support to cells during the hypothermic intervals of freezing and thawing, which dampens the induction of CIDOCD compared to traditional freezing media (Mathew et al., 1999; Baust et al., 2000, 2002). Several studies have reported improvements in cell viability, long-term survival and cell performance after use of CryoStor for the preservation of primary human immune and stem cells, neural progenitor cells, and iPSC-derived neurons (Stylianou et al., 2006; Clarke et al., 2009; Fortin et al., 2016; Seay et al., 2017; Wakeman et al., 2017). Our work provides the first application of CryoStor to primary rodent neuron cryopreservation, imparting an important new tool to neurobiology laboratories.

Using CS10, we achieved a viable cell yield of ∼58%. By comparison, the viable cell yield reported following cortical neuron cryostorage using traditional freezing media ranged from 10 to 30% (Higgins et al., 2011; Robert et al., 2016). Relative to a fresh dissection, our overall efficiency of cryopreservation is ∼69%. CS10 provides substantial improvements in long-term cell survival compared to other examined media, with an average reduction in survival of only 6%. In contrast, previous methods found, at best, a 40% reduction in long-term survival compared to freshly dissected controls (Mattson and Kater, 1988; Higgins et al., 2011). A recent publication using another pre-commercial, proprietary freezing media formulation also demonstrated improvements in cell viability and culture health compared to traditional freezing media (Pischedda et al., 2018). This underscores the importance of reagent choice when applying cryopreservation methodologies to primary neuron culture.

The percentage of recovered neurons relative to astrocytes was reduced in cryopreserved cultures, indicating that neurons are likely more sensitive to cryostorage than glia. Whether this sensitivity is induced by CS10 medium or a product of cryostorage per se was not investigated, although a demographic shift was also observed in cryostored cultures previously (Otto et al., 2003). Importantly, our results demonstrated that CS10-cryopreservation may be suitable for long-term storage of cortical neurons (more than one year) for certain applications, as well as the storage of hippocampal neurons. We believe this protocol represents a highly significant improvement on past methods and makes neuronal cryopreservation a feasible and efficient alternative to fresh dissections.

### Cryostored and freshly-obtained neurons are developmentally, morphologically, and functionally similar

The experimental utility of cryopreserved neurons depends on their ability to accurately replicate salient developmental events described in freshly dissected cells. Without exception, cultured neurons recovered from short-term cryostorage in CS10 could not be distinguished from those obtained from fresh dissection. It is important to note that the inability to detect a significant difference between CS10-cryostored and fresh neurons using the statistical methods and number of replicates presented in our study is not sufficient to conclude that the two populations are identical. Although comprehensive, our validation strategy is not exhaustive; therefore, we urge researchers to perform side-by-side validations of fresh and cryopreserved neurons to ensure equivalence in their most critical assays.

We assessed gene expression using a panel of markers for neurodevelopment and maturity, cortical neuron subtypes, and glutamatergic or GABAergic identity. No differential expression was detected between fresh and CS10-cryostored cortical or hippocampal cultures, or cultures recovered from three-month-old and one-year-old aliquots. However, four genes trended toward differential expression in individual experiments. *Tbr1* (Fig. 8*B*), *Slc17a7*, and *Mtap2* (Fig. 9*C*) were differentially expressed by less than two-fold, a value commonly cited as a rule-of-thumb threshold for considering a change potentially biologically meaningful. On the other hand, levels of *Reln*, a gene essential for neuronal migration and cortical layer formation *in vivo* (Santana and Marzolo, 2017), were reduced nearly five-fold in CS10-cryostored cortical cultures compared to fresh cultures. *Reln* levels could be reduced if a cue that stimulates its expression is weakened or absent after cryostorage, or due to reduced numbers of *Reln*-expressing cell types. Nonetheless, decreased *Reln* levels did not perturb axon outgrowth, synaptogenesis, or synaptic transmission, and therefore we are unable to comment on the biological relevance of this finding.

Neurons undergo extensive morphologic remodeling as they mature, which is essential for future connectivity. It is therefore critical that these events are unaffected by cryostorage. Our findings indicate that in cryopreserved cultures, morphogenesis and synapse formation occur in-step with freshly dissected controls, as no significant differences were found in the metrics measured in our studies at the examined timepoints. Fresh and cryostored neurons could not be discriminated by any morphologic variable, in agreement with previous studies of cortical neurons stored in traditional freezing media (Petite and Calvet, 1995). Additionally, in samples from the same source dissection, analyzed when freshly plated or when thawed at a later date, no morphologic differences could be detected.

Although synapse density was similar between fresh and cryostored neurons, the change in ratio of glutamatergic and GABAergic synapses in cortical neurons approached statistical significance. However, our RT-qPCR data failed to detect any cryostorage-dependent changes in *Slc17a7/6* (VGLUT1/2) or *Slc32a1* (VGAT) levels in cortical or hippocampal neurons, and PSD95-positive and gephyrin-positive synaptic connections were unperturbed in hippocampal neurons after cryostorage. Therefore, we conclude that following cryostorage, excitatory and inhibitory neurons were similarly preserved and capable of contributing to functional neuronal networks.

Cryopreserved dissociated cortical cultures contained a greater proportion of astrocytes relative to neurons. This demographic shift could alter paracrine support and neurotransmitter uptake, and thus influence neuron maturation and function (Bélanger and Magistretti, 2009). Indeed, Otto et al., postulated that an increase in astrocytes could account for the improved survival of cryopreserved neurons observed in their experiments (Otto et al., 2003). Depending on the goals of the experimenter, the presence of glia in primary neuronal cultures may be welcomed or a nuisance. Addition of the mitotic inhibitor AraC can prevent glial overgrowth in primary neural cultures (Kaech and Banker, 2006).

Using whole-cell patch clamp electrophysiology, we observed similar examples of spontaneous and evoked APs elicited by fresh and cryostored neurons, in agreement with previous reports that the number, burst duration, and amplitude of APs are not significantly affected following cryostorage in several media (Otto et al., 2003; Quasthoff et al., 2014; Pischedda et al., 2018). Additionally, careful analysis of specific features of evoked AP waveforms did not detect significant differences after recovery from cryostorage. Further population-wide details were extracted by investigating evoked responses to excitatory agonists, using calcium imaging and glutamate release assays. Stimulation of glutamate and glycine receptors, as well as depolarization-dependent Ca^+2^ channels, did not evoke differential responses between fresh and CS10-cryopreserved neurons. We infer that overall, synaptic organization and demographics are similar between fresh and CS10-cryopreserved neurons, and that these two populations are functionally indistinguishable.

While short-term cryostorage of neurons (three months) had essentially no deteriorating effects, cryostorage for one year affected some aspects of neuronal integrity. In particular, long-term cryostorage altered the rise time and half-width of evoked APs compared to short-term cryostorage. One-year cryostored neurons also displayed greater heterogeneity in these measurements. Cryostored cells in an academic laboratory are generally kept in communal liquid nitrogen storage dewars, and are exposed to frequent temperature changes as samples are added and removed. Indeed, two studies directly examined the viability, recovery, and function of primary human peripheral blood mononuclear cells after subjecting cryostored aliquots to temperature fluctuation cycles reminiscent of those caused by routine handling (Germann et al., 2013; Angel et al., 2016). Their work found that T-cell function was negatively affected by suboptimal storage. We did not control for handling and storage conditions in our experiments, which indeed is a critical component of cryopreservation methodologies. Whether our observed differences are a consequence of cryostorage itself or due to the accumulation of damage due to suboptimal storage conditions over time could not be tested by our current experimental design. These results underscore that care must be taken to preserve the integrity of cryostored neuron samples.

The ability to store and recover neurons with reliably high viability and fidelity has several benefits. (1) Cryopreservation reduces the use of animals. A cortical dissection can yield far more neurons than what is needed for a typical experiment. As surplus neurons are cryostored and no longer wasted, we have reduced our animal usage from several mice a month to several a year. (2) Cryopreserved neurons enable experimental flexibility. With neurons stockpiled, experiments can be plated as frequently as desired, a feat simply not practical when relying on timed pregnancies for source material. (3) Cryostored neurons are suitable for use in diverse experimental approaches and are amenable to genetic manipulations including viral transduction and electroporation (data not shown). Neuronal cryopreservation will likely be useful for labs regularly generating low density cultures for electrophysiology or imaging, and those with infrequent neuron experiments. This simple method has the potential to increase research productivity, as well as curtail the use of animals, while ensuring biological equivalence to the existing standard of experimentation.

## Synthesis

Reviewing Editor: Kirill Martemyanov, The Scripps Research Institute Decisions are customarily a result of the Reviewing Editor and the peer reviewers coming together and discussing their recommendations until a consensus is reached. When revisions are invited, a fact-based synthesis statement explaining their decision and outlining what is needed to prepare a revision will be listed below.

The authors have addressed all my previous concerns adequately in the current manuscript. With the added qPCR data, ephys data (with much-improved quality) and the revised glutamate release plots, the authors' claims on the cryopreserved neuron's properties are well supported.

I have only one comment on the PSD-95 labeling. It seems the PSD-95 labeling pattern in Fig. 6 is much more diffused than the staining patterns in Fig 9. Fig 9's staining looks more consistent with the known expression pattern of PSD-95 (concentrated on spine heads, less expression in dendrites and soma). Please address this discrepancy.

## Author Response

Response to the reviewers: We would like to thank the editor and the reviewers for their assessment of our study. We have revised our manuscript to address their concerns by including additional experiments and deeper discussion. Here, we provide a point-by-point response to their comments.

Remarks to the Author

Synthesis Statement

Both reviewers felt that the report contains potentially interesting information pertaining to new technique for neuronal cryopreservation. If such technique is indeed superior to currently used methods this could offer significant advantages for the field. However the reviewers also agreed that the manuscript in its present form contains many deficiencies and uncertainties that diminish the impact. In particular, two areas of concern include doubts about electrophysiological characterization and insufficient comparison of the technique relative to existing methods. Significant work would be required to address these concerns, and should this be possible to address we would be willing to consider the revised manuscript. The specific issues that need to be resolved are listed below.

We would like to thank the reviewers for acknowledging the potential significance of our method. In order to strengthen our study, we have done further characterization of the electrophysiological properties of cryostored neurons, as well as examined the effect of storage duration on performance, and characterized the efficacy of our method in the cryopreservation of hippocampal neurons.

Essential Comments

1. Electrophysiology: Authors should explain why some neurons show very broad action potential profiles with small amplitudes. Are these broad AP signals a mixture of APs and spontaneous EPSPs?

Pharmacological experiments using TTX and AMPAR, NMDAR antagonists will help to dissect the nature of this signal. Action potential recordings in Figure 6 are extremely noisy and seem to contain

digitized noise.

In Fig.6a and b, the action potentials have half-width &gt; 50 ms, and show a strange double peak profile. In addition, the bottom panel in Fig. 6a,

action potentials seem to have a size only around 20 mV and is inconsistent with authors claim that “spikes that overshot 0mV were used for experiments

and analysis” (Line 259). These action potential profiles are very atypical and thus raise the question of poor cell conditions. In figure 6a and 6b, the representative traces between the fresh and CS group do not look similar in their frequency, or dynamics of individual event (rise time, half width, amplitude). Before making the claim that neurons in the two preps are functionally indistinguishable, more individual traces need to be recorded, all of the above parameters need be quantified and compared.

We agree with the reviewer that our assessment of the electrophysiological properties of fresh and cryostored neurons was insufficient, and did not allow for convincing conclusions to be drawn regarding the equivalence of our cultures.
